# Culture Differences in Advance Care Planning and Implications for Social Work Practice

**DOI:** 10.1093/geroni/igab046.071

**Published:** 2021-12-17

**Authors:** Jung-Hwa Ha, Changsook Lee, Jennifer Yoo

**Affiliations:** 1 Seoul National University, Seoul, Seoul-t'ukpyolsi, Republic of Korea; 2 University of Georgia, Atlanta, Georgia, United States

## Abstract

Advance care planning (ACP) is the process of making plans and decisions regarding end-of-life care (EOLC) in advance while one has the physical and cognitive capacity to do so. However, even if health practitioners recognize the importance of ACP, they may be constrained by social and cultural factors in engaging their clients in ACP. This study examined cultural differences in ACP and various strategies that social workers use to initiate conversations on ACP in a range of settings. Using the case study method, we conducted in-depth interviews with 7 social workers who work in South Korea, 2 Korean-American social workers working in the Korean-American communities in the US, and 3 American social workers serving diverse populations in the US. Their practice sites include: university hospitals, day care centers, a community senior center, a nursing home, and a hospice agency. Social workers in both countries emphasized the need to build rapport with their clients early on and to empower them to take the lead in their ACP while they were still healthy. In Korean and Korean-American communities, social workers recognized their clients’ reluctance to speak about EOLC and highlighted the importance of communicating with their family due to their clients’ preference for family-centered decision-making. When doing this, a step-by-step approach in giving relevant information was recommended. We identified relationship-building, empowerment, and culturally sensitive approaches as common strategies in initiating discussions on ACP in both countries.

